# Infants Fed Breastmilk or 2′-FL Supplemented Formula Have Similar Systemic Levels of Microbiota-Derived Secondary Bile Acids

**DOI:** 10.3390/nu15102339

**Published:** 2023-05-17

**Authors:** David R. Hill, Rachael H. Buck

**Affiliations:** Abbott, Nutrition Division, Columbus, OH 43219, USA; david.hill1@abbott.com

**Keywords:** breastfeeding, human milk oligosaccharide, pediatric nutrition, metabolomics

## Abstract

Human milk represents an optimal source of nutrition during infancy. Milk also serves as a vehicle for the transfer of growth factors, commensal microbes, and prebiotic compounds to the immature gastrointestinal tract. These immunomodulatory and prebiotic functions of milk are increasingly appreciated as critical factors in the development of the infant gut and its associated microbial community. Advances in infant formula composition have sought to recapitulate some of the prebiotic and immunomodulatory functions of milk through human milk oligosaccharide (HMO) fortification, with the aim of promoting healthy development both within the gastrointestinal tract and systemically. Our objective was to investigate the effects of feeding formulas supplemented with the HMO 2′-fucosyllactose (2′-FL) on serum metabolite levels relative to breastfed infants. A prospective, randomized, double-blinded, controlled study of infant formulas (64.3 kcal/dL) fortified with varying levels of 2′-FL and galactooligosaccharides (GOS) was conducted [0.2 g/L 2′-FL + 2.2 g/L GOS; 1.0 g/L 2′-FL + 1.4 g/L GOS]. Healthy singleton infants age 0–5 days and with birth weight > 2490 g were enrolled (*n* = 201). Mothers chose to either exclusively formula-feed or breastfeed their infant from birth to 4 months of age. Blood samples were drawn from a subset of infants at 6 weeks of age (*n* = 35–40 per group). Plasma was evaluated by global metabolic profiling and compared to a breastfed reference group (HM) and a control formula (2.4 g/L GOS). Fortification of control infant formula with the HMO 2′-FL resulted in significant increases in serum metabolites derived from microbial activity in the gastrointestinal tract. Most notably, secondary bile acid production was broadly increased in a dose-dependent manner among infants receiving 2′-FL supplemented formula relative to the control formula. 2′-FL supplementation increased secondary bile acid production to levels associated with breastfeeding. Our data indicate that supplementation of infant formula with 2′-FL supports the production of secondary microbial metabolites at levels comparable to breastfed infants. Thus, dietary supplementation of HMO may have broad implications for the function of the gut microbiome in systemic metabolism. This trial was registered at with the U.S. National library of Medicine as NCT01808105.

## 1. Introduction

Medical consensus has long held that exclusive breastfeeding is the best source of nutrition and immune protection during early life. Exclusive breastfeeding through 12 months of age is strongly encouraged by prominent medical advisory groups including the American Academy of Pediatrics [1] and the World Health Organization [2]. However, access to healthcare, cultural expectations, and employment outside the home can present significant challenges to exclusive and prolonged breastfeeding [3]. In addition, there are uncommon but compelling instances in which breastfeeding may be contraindicated, such as in infants with significant metabolic disorders, in mothers undergoing radiotherapy, or taking certain pharmacologic agents that may be transferred through milk, the presence of some transmissible infectious disease, or in following mastectomy [1]. Therefore, the provision of nutritionally complete infant formula to support the developing immune system remains an essential goal for pediatric nutrition research.

Significant progress has been made in recent years regarding the fortification of some infant formulas with Human Milk Oligosaccharides (HMOs). HMOs are the 3rd largest solid component of human milk with a broad range of potential benefits, including prebiotic effects and enhanced gut maturation, gut motility, immunity, and cognition [4]. More than 150 unique HMO structures have been identified [5]. Although the individual composition is highly variable, 2′-FL is among the most abundant naturally occurring HMOs with levels ranging from 0.06–4.65 g/L in human milk [6]. Pioneering in vitro experiments indicated that 2′-FL inhibits the binding of gastrointestinal and respiratory pathogens [7,8,9] and further work in animal models has demonstrated that 2′-FL feeding also attenuates pro-inflammatory signaling within the gastrointestinal tract [10,11]. Epidemiologic studies have demonstrated that the presence of certain HMOs, including 2′-FL, in breastmilk is associated with protection from infectious disease [12] and that premature infants who carry the non-secretor trait have a greater risk of morbidity and mortality due to necrotizing enterocolitis [13].

Recent improvements in the efficiency of HMO synthesis have allowed for the production of HMOs on an industrial scale, making supplementation of infant formula with oligosaccharides that are chemically and structurally identical to those found in human milk more feasible [14,15]. Clinical studies have demonstrated that supplementation of infant formula with 2′-FL is well tolerated [16] and promotes systemic reduction in inflammatory cytokine levels relative to control formula and comparable to breastfed infants [17]. Additional data from the same patient cohort indicated that infants fed formula fortified with 2′-FL experienced fewer respiratory infections relative to infants fed control formula [18]. A similar study suggests that fortification of infant formula with 2′-FL and LNnT may be associated with lower rates of antibiotic use in young children [19].

HMOs, including 2′-FL, readily cross the epithelium of the gastrointestinal tract [20] and have been measured in both plasma and urine from breastfed infants [21,22,23]. HMOs and their secondary metabolites circulate systemically, affecting multiple organ systems and processes [24]. Given the known role of 2′-FL as a prebiotic capable of influencing the composition and metabolic function of the gut microbiome [25,26,27,28], we hypothesized that supplementation of 2′-FL may have substantial effects on the metabolic output of the intestinal microbiome, which contributes significantly to the systemic benefits associated with breastfeeding [29]. We conducted a prospective, randomized, and double-blinded study in a previously studied cohort [16,17] to examine circulating metabolite composition in infants fed one of several formula matrices differing only in their oligosaccharide content and relative to age-matched infants who were exclusively breastfed. We found differences in circulating metabolite composition between breastfed infants and those who were fed a control infant formula. Fortification of infant formula with 2′-FL was associated with an increase in the abundance of microbial fermentation products. We also observed that 2′-FL fortification resulted in a dose-dependent increase in the circulation of microbe-derived secondary bile acids. Secondary bile acids in circulation were associated with changes in circulating cytokine concentrations, suggesting changes to the systemic immune environment. Taken together, these results suggest that 2′-FL fortification of infant formula may support aspects of microbial metabolism typically associated with breastfeeding.

## 2. Materials and Methods

### 2.1. Study Design and Population

The present report is a new component of a previously described clinical study [16,17]. Blood samples were collected from a subset of healthy, full-term infants who participated in a prospective growth study, conducted at 28 sites throughout the United States from April 2013 through January 2014. Prior to enrollment, a parent or legally authorized representative of each enrolled infant gave written informed consent. The protocol, informed consent, and all study procedures were reviewed and approved by Schulman Institutional Review Board, Cincinnati, OH under the ethical approval code 201300836. This trial was registered at clinicaltrials.gov as NCT01808105.

- The inclusion criteria were as follows:
- singleton birth;
- gestational age 37 to 42 weeks;
- birth weight 2490 g;
- 0 and 5 days of age at enrollment;
- exclusive formula or breastfeeding since birth;
- overall good health in the infant’s medical history and parental report;
- A resident of a smoke-free home.

Exclusion criteria were the medical history of either the mother or child that might be considered to have potential developmental or growth effects including potential maternal substance abuse. Gestational diabetes was not considered exclusion criteria in cases where infant weight was within the 95th percentile [30]. The use of antibiotics was also considered a cause for exclusion, with the exception of routine antibiotic eye drops used at birth.

**Figure 1:**
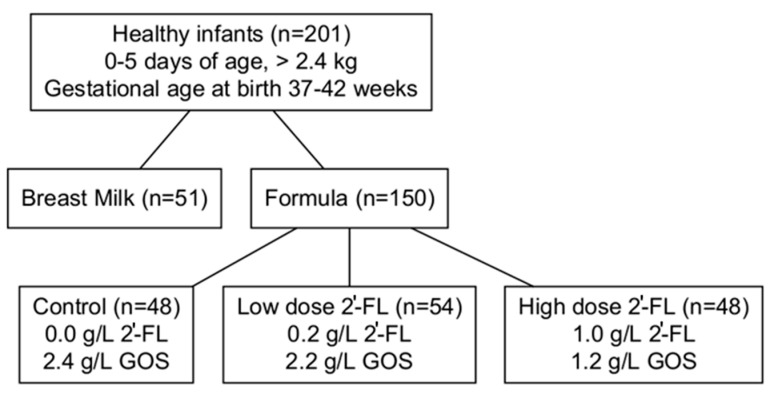
Flow diagram of participants recruited in this substudy. 2′-FL, 2′-fucosyllactose. GOS, galactooligosaccharides.

Infants whose parents intended to exclusively feed their infants formula were randomized to receive one of three formulas, all containing a total amount of 2.4 g/L of oligosaccharides, according to randomization schedules stratified by site and sex. A nonrandomized breastfed (BF) group was also enrolled. A flow diagram illustrating the study group assignments is given in Figure 1. The control formula (CF) contained galactooligosaccharides (GOS; Vivinal^®^ GOS, FrieslandCampina, Amersfoort, The Netherlands) only [CF: 0 g/L 2′-FL + 2.4 g/L GOS]; the experimental formulas (EFs) were fortified with varying levels of 2′-FL and GOS [EF1: 0.2 g/L 2′-FL + 2.2 g/L GOS; EF2: 1 g/L 2′-FL + 1.4 g/L GOS]. The three formulas contained 64.3 kcal/dL (19 kcal/fl oz) (Table 1).

”Exclusively fed” was defined as the sole use of breast milk or assigned formula, and no other liquids or solids. An exception was made for the use of non-antibiotic prescriptions, mineral supplements, and vitamins. Occasional use of alternative feedings, defined as less than 5 times during the study period, was considered consistent with exclusive feeding.

Participant selection for the substudy. Selection for the substudy was determined on the sole basis of parental or legal guardian authorization for the collection of blood samples. Participation in blood sample collection was entirely optional for all study participants

### 2.2. Anthropometric, Demographic Data, and Blood Sample Collection

At the enrollment visit, pre-study feeding regimens, birth anthropometric measurements, gestational age, and demographic data were collected, including race, number, and ages of siblings in the home, and mode of delivery. At 6 weeks (±3 days) of age parents were questioned to determine eligibility for optional blood sampling. The following exclusion criteria were applied:

- >240 mL per week of an alternate feeding other than the assigned study formula or >2 breastfeedings;
- Use of any alternate feeding in the preceding 48 h;
- Maternal or infant use of any oral anti-inflammatory medication.

### 2.3. Assays

#### 2.3.1. Metabolic Profiling

Sample Storage and Preparation. 2–3 mL of non-fasting venous blood was drawn into sodium heparin vacutainer tubes, shipped at ambient temperature to the laboratory, and received within 24 h of collection. Plasma was obtained by standard centrifugation procedure, dispensed into small plastic vials, and stored at −80 °C until analysis. Plasma samples were shipped frozen for analysis to Metabolon, Inc. (Durham, NC, USA). A total of 201 plasma samples were analyzed belonging to the treatment groups listed in Figure 1. Sample collection and analysis were both completed at the conclusion of the clinical study in 2014.

Metabolomic profiling was performed as described previously [31,32,33]. Metabolites were extracted from 100 µL plasma by the addition of cold methanol. The precipitated extract was split into five aliquots and dried under n_2_. The samples were re-suspended in platform-specific solutions before they were applied to the instruments. The untargeted metabolomic profiling platform employed for this analysis was based on a combination of four independent platforms: ultrahigh performance liquid chromatography/tandem mass spectrometry (UHPLC/MS/MS) optimized for basic species, UHPLC/MS/MS optimized for acidic species, UHPLC/MS/MS optimized for polar species and gas chromatography/mass spectrometry (GC/MS) [31,32,33]. LC-MS was performed on a Waters ACQUITY ultra-performance liquid chromatography (UPLC) and a Thermo Scientific Q-Exactive high resolution/accurate mass spectrometer interfaced with a heated electrospray ionization (HESI-II) source and Orbitrap mass analyzer operated at 35,000 mass resolution [31,32]. Samples destined for GC/MS analysis were derivatized under dried nitrogen using bistrimethyl-silyl-trifluoroacetamide (BSTFA). Samples were analyzed on a Thermo-Finnigan Trace DSQ fast-scanning single-quadrupole mass spectrometer using electron impact ionization at a unit mass resolution [33]. Metabolites were identified by matching the ions’ chromatographic retention index and mass spectral fragmentation signatures with reference library entries created from authentic standard metabolites. For ions that were not covered by the standards, additional library entries were added based on their unique retention time and ion signatures. Peak ion counts for each compound in each sample were used for statistical analysis, resulting in the comparisons of relative concentrations. A given compound was reported from only one of the four platforms.

#### 2.3.2. Targeted Assay of 2′-Fucosyllactose

2′-Fucosyllactose (2′-FL) was quantitated by LC-MS/MS. Human heparinized plasma was spiked with the internal standard, melezitose, and subjected to protein precipitation with methanol. After centrifugation, the organic supernatant was removed, evaporated, and reconstituted in Methanol/Water (75:25). An aliquot of the reconstituted extract was injected into Agilent 1290/AB Sciex QTrap 5500 LC-MS/MS system equipped with a BEH Amide normal phase UHPLC column. The peak area of the *m*/*z* 487→205 product ion of 2′-Fucosyllactose was measured against the peak area of the Internal Standard (Melezitose) production of *m*/*z* 503→323. Quantitation was performed using a weighted linear least squares regression analysis generated from freshly prepared calibration standards.

#### 2.3.3. Plasma Cytokine Measurements

Plasma cytokine levels were measured according to the methods previously published [17]. Among qualifying research subjects, 2–3 mL of non-fasting venous blood was drawn into sodium heparin tubes. Blood samples were shipped overnight to Lovelace Biomedical. Samples were transferred to frozen storage within 24 h of collection. Cytokine concentrations were measured in thawed plasma samples using a multiplex kit according to the manufacturer’s protocol (HCYTOMAG-60K-10, custom 10 analyte kit; EMD Millipore). 

### 2.4. Statistical Analysis

Demographic characteristics were analyzed using GraphPad Prism 6.02. Continuous values were compared by 1-factor ANOVA or Kruskal-Wallis test. Categorical variables were compared by χ^2^ and adjusted for multiple comparisons). All metabolomics analysis was conducted in R [34] using GNU Emacs v26.2 [35] on Windows 10. Plots were constructed using the R package ggplot2 [36]. Data analysis scripts documenting the complete statistical analysis are available by request from the authors.

## 3. Results

There were no significant differences among feeding groups for age at enrollment, gender, weight, length, incidence of Cesarian section, or head circumference at birth. There was a significant difference between the high dose 2′-FL and exclusive breastfeeding groups with respect to race, with the breastfed group having more infants that were white and the high dose 2′-FL formula fed having more infants that were black (Table 2). In the original study, 9 of the total 424 participants violated the criteria of “exclusively fed” (1 control formula, 3 low dose 2′-FL, 1 high dose 2′-FL, 4 breastfed) [16]. These numbers did not impact the mean birth weight of any of the groups.

### 3.1. Breastfeeding Is Associated with Significant Differences in Circulating Metabolites Relative to Control Formula

Although previous studies have examined the metabolic profiles of breastfed infants compared to formula-fed infants [37], improvements in HPLC/MS methodologies have expanded the range of measured metabolites. Thus we set out to broadly characterize the systemic metabolite profile of breastfed infants relative to formula-fed infants. Blood plasma was collected from all study participants at 6 weeks postpartum and blood metabolites were extracted for parallel analysis via GC/MS, LC/MS/MS, and Polar LC to ensure a broad range of metabolite identification. These measurements were matched to a library of metabolite standards for identification, and 743 out of 1113 metabolites were assigned chemical identities with a high degree of precision.

**Figure 2:**
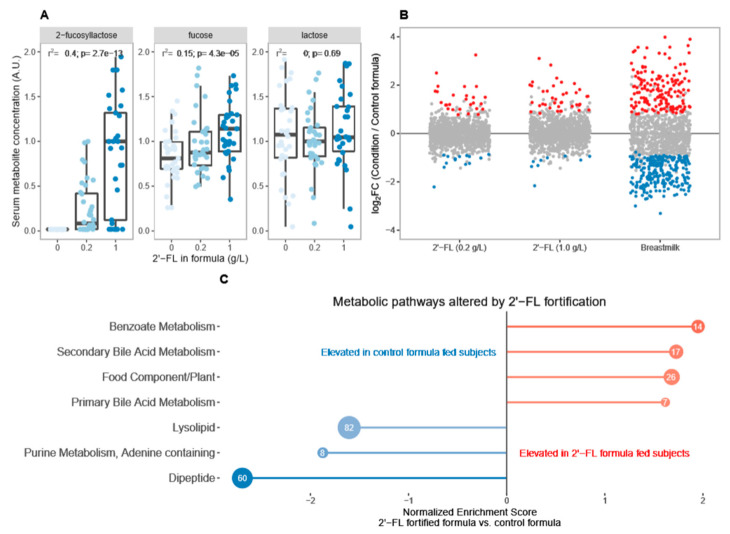
(**A**) Plasma 2′-FL levels are elevated in a dose-dependent manner among infants fed formula containing 0.2–1.0 g/L 2′-FL relative to infants fed formula without 2′-FL. Linear regression analysis demonstrated a strong correlation between formula 2′-FL concentration and relative abundance of 2′-FL (*r*^2^ = 0.4, *p* = 2.7 × 10^−13^) and fucose (*r*^2^ = 0.15, *p* = 4.3 × 10^−5^) in the plasma but was not correlated with plasma lactose concentration. (**B**) Differentially expressed plasma metabolites in infants fed formula containing 0.2–1 g/L 2′-FL or breastfed infants relative to infants fed control formula that did not contain 2′-FL. Red indicates metabolites that are significantly increased relative to infants fed control infant formula. Blue indicates metabolites that are significantly lower relative to infants fed control infant formula. (**C**) GSEA analysis was conducted using a list of all significantly different metabolites ranked according to the average log2-transformed fold change in 2′-FL fed infants relative to infants fed control formula. This ranked list was evaluated for the enrichment of metabolites in known KEGG metabolic pathways and a NES was computed to evaluate the relative enrichment of metabolites in 2′-FL fed infants relative to infants fed control formula. NES > 0 indicates relative enrichment in infants fed formula containing any amount of 2′-FL and NES < 0 indicates relative enrichment among infants fed control formula relative to 2′-FL enriched formula. The number of metabolites in each pathway is indicated in the bubble. Only pathways with statistically significant (*p* < 0.05) NES are shown.

To better understand the differences in systemic metabolites between breastfed and infants fed control formula, we computed the log_2_-transformed fold change in the relative abundance of 1113 metabolites among all breastfed infants relative to all infants fed control formula at 6 weeks postpartum and utilized a two-tailed Wald test [38] to evaluate the statistical significance of the observed differences (*p* < 0.05). This analysis resulted in 178 metabolites that were significantly higher among breastfed infants and 238 metabolites that were significantly lower relative to infants fed the control formula (Figure 2, Supplemental Table S1). These analyses revealed differences in systemic metabolites between breastfed infants and infants who were exclusively fed control infant formula.

### 3.2. Fortification of Formula with 2′-FL Increases Levels of Circulating Microbiota-Derived Metabolites

Given that our data demonstrated significant differences in systemic metabolites in breastfed infants relative to infants who were exclusively formula fed, we asked whether targeted fortification of infant formula could be utilized to support a metabolic profile that more closely resembled that seen among breastfed infants. HMOs, and 2′-FL in particular, have been established as potent prebiotic compounds both in vitro [24,25,26] and in vivo [19]. We hypothesized that fortification of infant formula with prebiotic 2′-FL might result in emergent effects on systemic metabolites due to its influence on the composition of the developing microbiota, and the subsequent influence of those microorganisms on the metabolic activity of the gut.

We examined plasma metabolite composition among infants fed formula fortified with 2′-FL at 0.2 g/L (*n* = 36) or 1.0 g/L (*n* = 35) relative to matched infants who were fed control formula without 2′-FL (*n* = 37) or who were exclusively breastfed (*n* = 40) at 6 weeks postpartum. Analysis of plasma 2′-FL levels revealed a linear correlation with the level of 2′-FL fortification among formula-fed infants (Figure 2A, *r*^2^ = 0.4, *p* = 2.7 × 10^−13^), indicating that 2′-FL was absorbed into the circulation in proportion with the dietary intake. Similarly, plasma fucose levels were correlated with 2′-FL supplementation in a dose-dependent manner among formula-fed infants (Figure 2A, *r*^2^ = 0.15, *p* = 2.743 × 10^−5^). 2′-FL fortification was not associated with any change in plasma lactose concentrations in formula-fed infants.

However, the potential impact of 2′-FL fortification on plasma metabolites extends well beyond the increase in 2′-FL and its immediate catabolic products, fucose, and lactose. We examined the fold change in plasma metabolites in infants fed formula containing 0.2–1.0 g/L 2′-FL relative to infants fed control formula (Figure 2B). In comparison to the metabolic changes associated with breastfeeding relative to the control formula, the impact of 2′-FL fortification alone is substantial and accounts for 48 differentially abundant plasma metabolites. Gene Set Enrichment Analysis (GSEA) [39] revealed that these differences in plasma metabolites were consistent with a general increase in markers of benzoate metabolism, a process associated with many dairy products [40], and bile acid metabolism (Figure 2C). Markers of dipeptide metabolism, purine metabolism, and lysophospholipid metabolism were somewhat lower among infants fed 2′-FL-containing formula relative to breastfed infants fed control formula.

**Figure 3:**
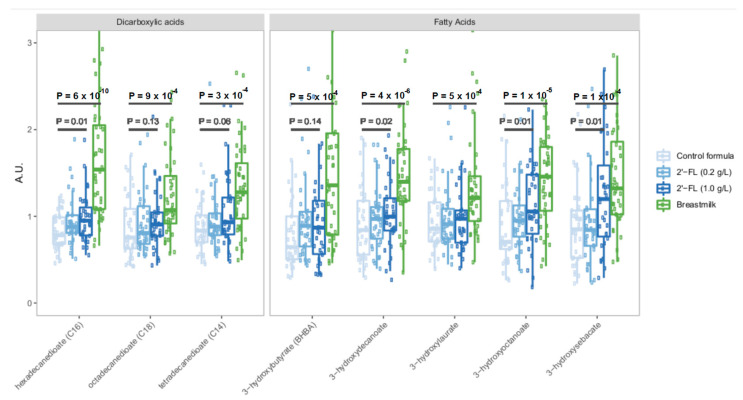
Relative levels of key microbial metabolites dicarboxylic acids, medium- and short-chain fatty acids in each study group. Statistical results represent a one-tailed Wilcoxon ranked-sum test for the comparisons indicated by black bars.

Given prior data establishing 2′-FL as a potent and selective prebiotic oligosaccharide [24,25,26], we examined broad changes in bacterial fermentation products in infants fed formula containing 2′-FL, infants fed control formula, and infants who were exclusively breastfed (Figure 3). This analysis revealed increases in microbial fermentation products such as dicarboxylic acids, and both medium- and short-chain fatty acids (SCFA) that were associated with the level of 2′-FL fortification (Figure 3). Remarkably, some SCFAs were present in 2′-FL fed infants at levels comparable to the levels in breastfed infants. We also noted an apparent trend towards dose-dependent increases in microbial fermentation products, with 1 g/L 2′-FL fortification supporting higher levels of some microbial metabolites relative to infants fed formula containing 0.2 g/L of 2′-FL.

### 3.3. Levels of Circulating Secondary Bile Acids Differ Significantly in Breastfed Infants Compared to Infants Fed Control Formula

**Figure 4:**
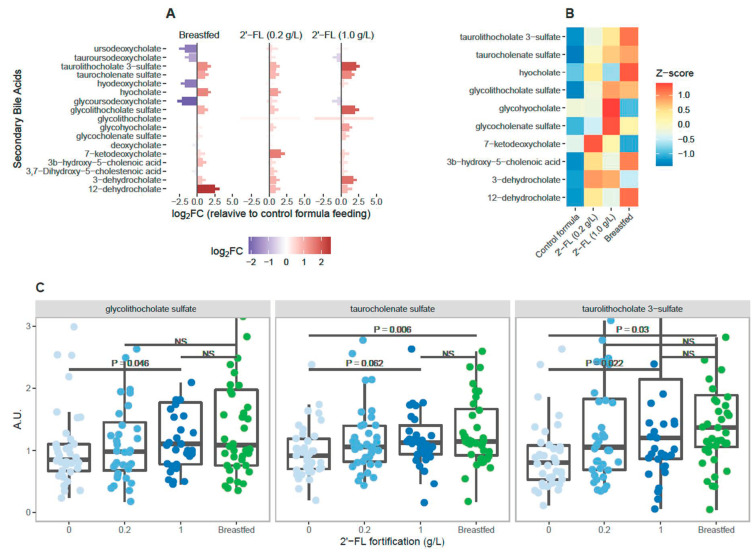
(**A**) Mean log2-transformed fold change in secondary bile acids detected in breastfed infants relative to infants fed control formula that did not contain 2′-FL. Red indicates bile acids that are higher relative to infants fed control formula. Blue indicates metabolites that are lower relative to infants fed control infant formula. (**B**) Heat map showing normalized Z-scores for plasma secondary bile acids among infants fed control formula, formula containing 0.2–1.0 g/L 2′-FL, or infants who were exclusively breastfed. (**C**) Box-plot showing relative abundance of key secondary bile acids in plasma among formula fed infants at two levels of 2′-FL fortification and in the plasma of breastfed infants. Statistical results represent a one-tailed Student’s *t*-test for the comparisons indicated by black bars.

Significant metabolic differences associated with the 2′-FL fortification of infant for- mula occur in processes associated with or dependent upon the intestinal microbiota (Figure 2 and Figure 3). The most notable among these differences are the changes we observed in secondary bile acid metabolism. Secondary bile acids are exclusively generated by the intestinal microbiota through the action of bile salt hydrolases that convert a limited pool of primary bile acids produced in the liver into a highly heterogeneous mix of derivative structures [41,42]. In the gut, secondary bile acids emulsify dietary lipids [42], suppress the growth of pathogenic microbes [43,44], and promote epithelial cell renewal [45]. The majority of bile acids are reabsorbed by the small intestine and circulate systemically, where they regulate immune homeostasis [46]. Given the importance of secondary bile acids to human health, we first wanted to understand how the bile acid profile of breastfed infants might differ from the bile acids circulating within infants fed control infant formula. We found that many secondary bile acids differed between breastfed infants and infants fed control formula, including 12-dehydrocholate, hyocholate, tau- rolithocholate 3-sulfate, taurocholenate sulfate, and glycolithocholate sulfate which were higher in the plasma of breastfed infants relative to infants fed control formula (Figure 4A). Other secondary bile acids, such as glycoursodeoxycholate, hyodeoxycholate, and ursodeoxycholate were more abundant in control formula-fed infants in comparison to breastfed infants.

### 3.4. 2′-FL Fortification Is Associated with Dose-Dependent Increase in Circulating Secondary Bile Acids

We next examined the dose-dependent influence of 2′-FL fortification on plasma secondary bile acid levels relative to control formula feeding or breastfeeding (Figure 4B,C). Here it became apparent that 2′-FL fortification of infant formula changes the levels of many secondary bile acids in plasma to levels similar to those seen among breastfed infants, as shown in Figure 4B. The concentration of secondary bile acids in plasma was clearly dependent upon the level of 2′-FL fortification in infant formula (Figure 4C). Thus, 2′-FL fortification impacts aspects of microbial metabolism in the gut compared to control infant formula and increases the production of key bile acid metabolites that may be involved in systemic immune homeostasis.

### 3.5. Elevated Secondary Bile Acids Are Correlated with Immunoregulatory Cytokine Levels in Plasma

**Figure 5:**
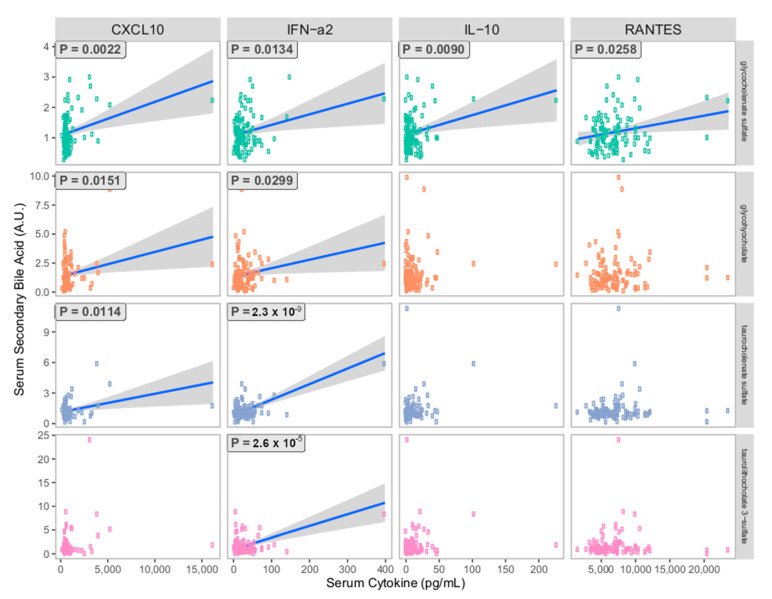
Linear regression analysis comparing plasma secondary bile acid levels and plasma cytokine concentrations among all study participants at 6 weeks postpartum. A *p*-value is given for combinations that meet the level of statistical significance (*p* < 0.05). Additional cytokines evaluated but not found to have any statistically significant correlation to plasma secondary bile acids include: interferon-*γ*, IL-1ra, IL-1*α*, IL-1*β*, and TNF-*α*.

Given the potential significance of changes in secondary bile acid metabolism for systemic immunity, we hypothesized that secondary bile acid metabolism might be associated with changes in immune mediators in our study participants. The study design included the measurement of plasma cytokine levels in a portion of the blood drawn at 6 weeks post-partum. Notably, these were the same blood samples used to measure plasma metabolites for our other analyses. The following cytokines were measured in plasma via ELISA in all study participants: interferon-*α*2, interferon-*γ*, Interleukin 10 (IL-10), Interleukin-1 receptor antagonist (IL-1ra), Interleukin-1*α* (IL-1*α*), Interleukin-1*β* (IL-1*β*), Interleukin-6 (IL-6), C-X-C motif chemokine ligand 10 (CXCL10, i.e., IP-10), RANTES i.e., CCL5, and tumor necrosis factor *α* (TNF-*α*). We then applied a linear regression analysis to examine the potential correlation between secondary bile acid levels in plasma and plasma cytokine levels across all study participants (Figure 5). We found that plasma levels of the secondary bile acid glychocholenate sulfate were significantly and positively correlated with concentrations of the cytokines interferon-*α*2, IL-10, RANTES, and the chemokine CXCL10. Other secondary bile acid structures, including glycohyocholate, taurocholenate sulfate, and taurolithocholenate 3-sulfate exhibited significant and positive statistical correlations with plasma concentrations of CXCL10 and interferon-*α*2. These observations suggest that increased circulation of secondary bile acids may promote certain aspects of systemic immune signaling involved in antigen recognition and immune cell trafficking.

## 4. Discussion

In the present study, we characterized circulating plasma metabolites in breastfed infants relative to infants who were fed one of multiple formula preparations that differed only in their oligosaccharide content. Our data demonstrate that there are multiple differences in circulating metabolites between breastfed infants and infants who were exclusively fed control infant formula (Figure 2). Fortification of infant formula with 2′-FL was associated with an increase in the production of circulating metabolites derived from gut microbial metabolism (Figure 2, Figure 3, Figure 4 and Figure 5). In particular, 2′-FL fortification resulted in a dose-dependent increase in circulating levels of secondary bile acids relative to the control formula and reaching concentrations comparable to our observations in breastfed infants (Figure 4). Previous reports have demonstrated that secondary bile acid metabolism regulates systemic immunity [46,47] and in the present study, secondary bile acid metabolites in plasma were correlated with plasma cytokine and chemokine concentrations (Figure 5). To the best of our knowledge, these findings represent the first report linking human milk oligosaccharide consumption to the production of secondary bile acids by the gut microbiota in infants.

Metabolomics is a growing field of research utilizing high throughput liquid and gas chromatography, mass spectrometry, and nuclear magnetic resonance techniques to independently quantify and analyze vast numbers of unique metabolites in biological samples [48]. Previous studies have compiled broad surveys of the metabolites present in breastmilk itself [49,50] as well as the differences in circulating metabolites between breastfed and formula-fed infants [37]. Studies examining the differences between formula-fed and breastfed infants have utilized a variety of techniques to measure 356 metabolite composition in stool [51,52], urine [21,52,53,54], and plasma [21,55,56] samples collected from infants aged 0–24 months. However, wide variations in methodology, sample collection, and study populations have limited the generalizability and reproducibility of these studies [37]. To the best of our knowledge, the present study represents one of the largest surveys of the infant metabolome with 200 enrolled participants. This dataset also includes a wider survey of metabolites (743 known metabolites) than any of the previous reports (<200) [37]. The results are broadly consistent with previous reports associating variation in amino acid metabolism [37] and lipid metabolism [51] with the mode of feeding (Figure 2).

Importantly, the large study cohort and range of metabolites measured have facilitated insights into the metabolic differences between the metabolism of breastfed and formula-fed infants. Given the large number of differentially expressed metabolites that we observed, we applied Gene Set Enrichment Analysis (GSEA) [39] to evaluate the potential for coordinated shifts in metabolites that might be influenced by the mode of feeding. This analysis confirmed previous reports [37,51,55,57] suggesting that steroid metabolism, lipid metabolism and biosynthesis, and amino acid metabolism vary somewhat according to the mode of feeding (Figure 2C). These data characterizing changes in metabolites according to the mode of feeding provide a basis against which to evaluate the benefits of changes to infant formula feeding regimens.

The addition of human milk oligosaccharides (HMOs) is perhaps the most significant recent innovation in infant formula. New methods of production have enabled the cost-effective fortification of some infant formulas with oligosaccharides that are biochemically identical to those that occur in nature [14,15]. Fortification of infant formula with 2′-FL is safe [16,18] and has been shown to confer important benefits to immunity [17] and gut health [19]. Pre-clinical data suggest additional benefits of 2′-FL, which serves as a potent prebiotic [24,25,26,28] and anti-inflammatory immune modulator [10,11,58,59,60,61]. We hypothesized that 2′-FL fortification of control infant formula might alter some systemic metabolic markers and recapitulate metabolic features associated with the consumption of HMOs during breastfeeding. Previous reports have demonstrated that 2′-FL is present in the plasma of breastfed infants at levels that correlate with the concentrations found in the corresponding breast milk; 2′-FL was not detected in plasma samples from infants fed control formula [21]. Our results are entirely consistent with those of Goerhing et al. [21] in that infants fed control formula do not have detectable levels of 2′-FL in the circulation. However, our results also demonstrate that the fortification of infant formula with 2′-FL resulted in a highly significant dose-dependent increase in circulating 2′-FL and free fucose (Figure 2A). This offers additional evidence that dietary 2′-FL is readily absorbed in the GI tract and circulates systemically where it may exert immunoregulatory benefits [24].

The differences in metabolite profiles between infants fed control formula and those fed 2′-FL fortified formula extended beyond 2′-FL and its immediate catabolic products fucose and lactose (Figure 2B). This suggested that the addition of 2′-FL might support additional metabolic activity. Given the known role of 2′-FL as a potent prebiotic and substrate for fermentation by the gut microbiota [24,25,26,28], we examined the levels of multiple bacterial fermentation products in formula-fed and breastfed infants (Figure 3). 2′-FL fortification was associated with elevated levels of dicarboxylic acids, and medium and short-chain fatty acids typically associated with bacterial fermentation. In some cases, 2′-FL fortification resulted in the production of these metabolites at levels comparable to our observations in breastfed infants. This suggested that dietary 2′-FL acts as either a direct substrate for bacterial fermentation or that it supports the growth of strains that are participating in the fermentation of other dietary compounds.

Other metabolites associated with microbial activity were elevated in infants fed 2′-FL fortified formula. We applied GSEA to analyze the coordinated regulation of metabolites within known metabolic pathways and found that 2′-FL fortification was associated with a significant increase in bile acid metabolism (Figure 2C). Primary bile acids are synthesized from cholesterol in the liver and released into the duodenum via the gallbladder. They play an essential role in the emulsification of dietary lipids, allowing for the efficient absorption of fats in the proximal bowel [62]. At least 95% of bile acids released into the intestinal lumen are reabsorbed by the intestinal epithelium and enter the circulation where they can be reduced to cholesterol and recycled by hepatocytes [63]. This includes a diverse pool of structures known as secondary bile acids. These are generated through a two-step process that involves first the deconjugation of glycine or taurine by intestinal microbes expressing bile salt hydrolases followed by a wide variety of dihydroxylation reactions that result in structural heterogeneity [41,42]. Mode of feeding in infancy has been associated with changes in lipid metabolism [37] and there is some preclinical evidence to suggest that at least one bile acid, cholic acid, is altered in formula-fed primates [64].

We conducted an extensive characterization of differentially expressed secondary bile acids in breastfed infants relative to infants fed the control formula (Figure 4A). Several secondary bile acids, notably 12-dehydrocholate, hyocholate, taurolithocholate 3-sulfate, taurocholenate sulfate, and glycolithocholate sulfate were significantly higher in the circulation of breastfed infants relative to control formula-fed infants. A smaller subset of deoxycholate structures were elevated in control formula-fed infants relative to breastfed infants (Figure 4A). These results indicate that the composition of the circulating pool of secondary bile acids is dependent upon the mode of feeding in infants, a finding that has recently been independently observed in stool samples by Sillner et al. [65]. Upon further examination, it became clear that fortification of infant formula with 2′-FL resulted in the increased abundance in plasma of many of the secondary bile acids associated with breastfeeding (Figure 5B). In many cases, this relationship was dose-dependent with higher levels of 2′-FL fortification being correlated with higher levels of circulating secondary bile acids (Figure 5C). Thus, 2′-FL fortification of infant formula supports the generation of secondary bile acid metabolites at levels typically associated with breastfed infants.

The absence of data on the composition of the fecal microbiota and fecal secondary bile acids in these study participants is a significant limitation of this study. Given the known prebiotic activity of HMOs and 2′-FL in particular [24,25,26,28], it is possible that the changes in secondary bile acid levels associated with 2′-FL feeding are caused by changes in the composition of the microbiota. Alternatively, 2′-FL may activate alternate metabolic activity by the existing microbiota. Finally, differences in intestinal absorption of secondary bile acids under different feeding conditions cannot be ruled out as a contributing factor in the observed differences in plasma secondary bile acids.

The physiologic significance of secondary bile acids has undergone significant reevaluation in recent years. Emerging pre-clinical studies have demonstrated that these microbe-derived metabolites play a key role in regulating systemic immunity [41,42,47,62]. Secondary bile acids are innate ligands of G protein-coupled bile acid receptor 1 (GPBAR1) and Farnesoid X-Receptor (FXR), expressed by intestinal epithelial cells, and macrophages, dendritic cells, and natural killer T-cells found throughout the gastrointestinal-associated lymphoid tissue (GALT) [66]. Activation of these pathways by secondary bile acids is broadly anti-inflammatory, inhibiting NF-*κ*B mediated cytokine secretion and NLPR3 Inflammasome activity [66,67,68,69]. Dysbiosis, or the dysfunctional composition of the intestinal microbiota, alters the pool of available secondary bile acids [41,42,70] and may result in increased susceptibility to inflammatory disease and infection [43,71,72,73]. Recently, secondary bile acids have been shown to play an essential role in the development of immune homeostasis through the proliferation of a robust population of anti-inflammatory ROR*γ*^+^ T regulatory cells within the GI tract [46]. We hypothesized that the changes in circulating secondary bile acids that we observed across our infant feeding cohort might be associated with systemic changes in immune function. We identified multiple secondary bile acids that were positively correlated with concentrations of immune modulators in plasma (Figure 5). In particular, increased plasma levels of glycocholenate sulfate were associated with elevated levels of CXCL10, interferon-*α*, interleukin-10, and RANTES. This profile may be consistent with a state of gut immune homeostasis associated with robust secondary bile acid signaling [46,62,66,72]. For example, CXCL10 and IFN-*α* play an important role in the formation of long-lived memory T-cell populations which are critical to immune homeostasis [74,75]. IL-10 is a well-characterized mediator of anti-inflammatory regulatory T cell function [76,77]. RANTES (CCL5) is a multifunctional chemotactic cytokine that serves as one of the key signals for regulatory T cell homing [78]. Future studies may investigate whether the restoration of secondary bile acid metabolites through the fortification of infant formula with 2′-FL could mitigate the risk of allergy, autoimmunity, or inflammatory disease among formula-fed infants.

In summary, the present study demonstrates that fortification of infant formula with the HMO 2′-FL results in the dose-dependent restoration of certain metabolites derived from microbial activity in the gastrointestinal tract, particularly secondary bile acids. These secondary bile acids play a putative role in the development and maintenance of immune homeostasis [41,42,46,47,62] and our data supports a correlation between increased levels of circulating secondary bile acids and the activation of systemic immune mediators.

## Figures and Tables

**Table 1:** Composition of study formulas. GOS, galactooligosaccharides.

Ingredient	Control Formula	Low Dose 2′-FL	High Dose 2′-FL
Energy, kcal/dL	64.3	64.3	64.3
Protein, g/L	13.3	13.3	13.3
Fat, g/L	34.7	34.7	34.7
Total carbohydrate, g/L	69.0	69.0	69.0
GOS, g/L	2.4	2.2	1.4
2′-FL, g/L	0.0	0.2	1.0

**Table 2:** Baseline characteristics of infants enrolled in this sub-study. Values are means *±* SEM or mean with percentages of the total in parentheses. 2′-FL, 2′-fucosyllactose. Weight, length, and head circumference were measured at birth. Data on Weight, Length, and head circumference are stratified by gender. Continuous variables were compared between study groups by ANOVA. Categorical variables were compared by Chi-squared test according to the methods described above.

	Control Formula (*n* = 48)	Breastfed (*n* = 51)	Low Dose 2’-FL (*n* = 54)	High Dose 2’-FL (*n* = 48)	*p*
Age at enrollment, days	38.1 ± 0.1	3.5 ± 0.2	3.4 ± 0.2	3.8 ± 0.2	0.30
Males, *n* (%)	27 (56)	31 (61)	24 (44)	23 (48)	0.32
Gestational age, weeks	39.3 ± 0.2	39.4 ±0.1	39.2 ± 0.1	39.4 ± 0.2	0.51
Weight, g					
Male	3338 ± 70	3498 ± 92	3248 ±75	3322 ± 86	0.17
Female	3269 ± 94	3354 ±78	3188 ± 83	3191 ± 69	0.27
Length, cm					
Male	50.5 ± 0.3	51.2 ±0.4	50.5 ± 0.4	51.2 ± 0.5	0.32
Female	50.6 ± 0.3	50.9 ± 0.6	49.7 ± 0.4	50.1 ± 0.4	0.26
Head circumference, cm					
Male	34.8 ± 0.5	35.2 ± 0.4	34.5 ± 0.4	34.4 ± 0.5	0.49
Female	34.1 ± 0.4	33.9 ± 0.5	33.2 ± 0.4	33.5 ± 0.4	0.44
Race, *n* (%)					0.02
White	30 (63)	35 (69)	29 (54)	20 (42)	
Black	12 (25)	7 (14)	12 (22)	21 (44)	
Other	6 (13)	9 (18)	13 (24)	7 (15)	
Mode of delivery, *n* (%)					0.58
Vaginal	30 (63)	38 (75)	38 (70)	35 (73)	
C-Section	18 (38)	13 (25)	16 (30)	12 (27)	
Siblings in home	1.3 ± 0.2	1.2 ± 0.2	1.5 ± 0.2	1.2 ± 0.2	0.55

## Data Availability

The data presented in this study are available on request from the corresponding author.

## Supplementary Materials

The following supporting information can be downloaded at: https://www.mdpi.com/article/10.3390/nu15102339/s1, Supplementary Table S1: Differentially expressed metabolites.
